# Reference range of zinc in adult population (20-29 years) of Lahore, Pakistan

**DOI:** 10.12669/pjms.303.4027

**Published:** 2014

**Authors:** Warda Hussain, Asim Mumtaz, Farzana Yasmeen, Sana Qayyum Khan, Toqeer Butt

**Affiliations:** 1 Warda Hussain, University of Health Sciences, Lahore, Pakistan. Shalamar Medical & Dental College, Lahore, Pakistan.; 2 Asim Mumtaz, Shalamar Medical & Dental College, Lahore, Pakistan.; 3 Farzana Yasmeen, Shalamar Medical & Dental College, Lahore, Pakistan.; 4 Sana Qayyum Khan, Shalamar Medical & Dental College, Lahore, Pakistan.; 5 Toqeer Butt, Shalamar Medical & Dental College, Lahore, Pakistan.

**Keywords:** Atomic absorption spectrometery, IFCC, Reference range, Serum zinc

## Abstract

***Objective:*** To determine the reference range of zinc in adult population (age range 20 to 29 years) of Lahore.

***Methods:*** It was a descriptive cross sectional study which was carried out from Jan- August 2012 in Chemical Pathology Department of University of Health Sciences, Lahore. Serum zinc concentration was measured by flame atomic absorption spectrometry in randomly selected 450 healthy adults aged 20 to 29 years. After application of exclusion criteria reference values were determined in apparently healthy subjects according to guidelines of International Federation of Clinical Chemistry. The data was entered & analyzed using SPSS version 20.0. Serum Zn levels was expressed as Mean ± SD.

***Results:*** A total of 450 healthy subjects were included in this study. Out of these, 234 were females. Mean age was 25±0.13years. The mean concentration of zinc in serum of healthy individual was 24.02±7.03 µmol/L (range11.47-36.72). The mean±SD for males subjects were 22.33±6.42 µmol/L(range11.93-32.4). Similarly the mean±SD for females were 21.72±7.34 µmol/L (range9.94-36.87).

***Conclusion:*** This study presents reference range for serum zinc concentration in adult population of Lahore. The results showed that there is significant difference in serum level of zinc among different countries. This study will help us in establishing reference ranges of trace elements on larger population in future.

## INTRODUCTION

The availability of accurate trace element reference values in human is an important indicator to the health status of the general population. It can also be used for the different occupational groups which are under the continuous exposure of these trace elements.^1^ Most of the available reference intervals for laboratory tests were determined over two decades ago on older instruments and technologies, and they are no longer relevant considering the current testing technologies which are used by clinical laboratories. It is thus critical and of utmost urgency that a more acceptable and comprehensive database should be established.^2^ The role of the laboratory scientist is to help the clinician in interpreting observed values, by providing relevant reference values in a convenient and practical form.^3^ In Pakistan, reference values established in the western population are used, but these can be questioned due to differences in genetic load, lifestyle, and diet.^4^

Zinc (Zn) is an essential trace element. It is called as essential because it is involved in multiple biological processes.^5^ In order to assess the relation of this trace element with different diseases, it is important to have a baseline status of trace elements in the body. So this requires establishment of accurate reliable reference values that can be used as a tool in clinical decision.^6^

One of the essential trace elements in the body is Zn. 80% of the RBCs are mainly composed of Zn. The storage house for Zn is in muscles. 80% 0f plasma Zn is attached to the albumin rest of which is bound to alpha-2 macroglobin. Only a small concentration of Zn is present in amino acids.^7^ Zn is widely distributed in food and is readily obtainable from meats, whole grains, legumes, sea-food, poultry, cereals and pork.^8^ In United States, the dietary reference intake (DRI) for Zn is 11mg/day for male and 8mg/day for female.^9^

Zn with vitamin A participates in many body functions. It is involved in maintenance of immune function in humans.^10^ Zn being a component of more than 200 enzymes plays an important role in the body and it forms part of the structural protein e.g. the zinc finger.^11^

The objective of this study was to establish reference range of Zn in population of Lahore aged 20 to 29 years.

## METHODS

 A descriptive cross sectional study was conducted in the Department of Chemical Pathology, University of Health Sciences, Lahore. Four hundred fifty healthy individuals were included in the study based on IFCC & CLSI C28-P3 criteria Defining, establishing & Verifying reference interval in the clinical laboratory; Proposed Guidelines, 2008.They were recruited from nine different zones of Lahore with age ranging from 20 to 29 years and were randomly selected. The study was approved by the ethical committee of University of Health Sciences, Lahore.

These healthy subjects (20-29 years) were selected from adult population of Lahore based on inclusion criteria from 9 zones of Lahore. Five union councils were selected randomly from each zone. From each selected union council ten healthy subjects who were assessed clinically were selected for the study. So a total of 450 samples were collected from randomly selected 45 union councils. All demographic details were entered in the Proforma.

Informed consents were taken from the individuals participating in the study. The samples were collected under aseptic conditions. About 7ml of blood was collected in 2 serum separator vacutainer tubes (BD vacutainer SST) one for the determination of serum zinc and other 3.5 ml blood was collected in SST for determination of albumin, glucose & ALT. Urine sample was also collected. The sample was transported to University of Health Sciences, Lahore in an ice box containing ice bags. Serum was separated by centrifugation at 3000 g for 10 minutes.

Serum was shifted into 1ml nitric acid treated aliquot (for trace elements determination) & the other separated serum was collected in a separate aliquot for ALT, albumin & sugar levels. Aliquots were properly labeled according to the numbers on the Proforma of the patients. Urine examination for protein & sugar was also performed.

Serum glucose was measured by end point method on Selectra (Merck). Albumin was performed by colorimetric (Bromocresol green) method on Selectra (Merck). Serum ALT was determined by photometric method on Selectra (Merck). Urinary proteins & sugar were determined by Combur10 Test strips by Cobas. Zn was measured on atomic absorption spectrophotometer (Hitachi Z-2000). Calibration was done by ICP multi-element standard solution IV CertiPUR (Lot no: HC895244) at 5 different concentrations by serial dilution.

The data was entered & analyzed using SPSS version 20.0 (SPSS Inc., Chicago). Serum Zn levels was expressed as Mean±SD.

## RESULTS

Four hundred fifty healthy subjects (216 males & 234 females) with age ranging from 20 to 29 years who fulfilled the inclusion criteria were recruited from 9 different zones of Lahore. Their health status was assessed after detailed history & physical examination. Blood sugar random, ALT, Albumin, urine for proteins & sugar were performed. The results of these tests were also in the normal range. Serum zinc was estimated on blood samples of these healthy subjects. Serum Zn was estimated on atomic absorption spectrometer which is the reference method for the determination of trace elements.

 Zinc in the current study had a mean concentration of 24.02±7.03 µmol/L (range11.47- 36.72). The mean±SD for males subjects were 22.33±6.42 µmol/L (range11.93-32.4). Similarly the mean±SD for females were 21.72±7.34 (range 9.94-36.87). Detailed results are shown in Table-I.

**Table-I:** Minimum & Maximum level of ZINC in µmol/L along with 95^th^ confidence interval (CI), also the 2.5 percentile & 97.5 percentile

*Zinc*	*Min* *µmol/L*	*Max* *µmol/L*	*95* ^th ^ *CI*	*2.5* ^th^ *Percentile*	*97.5* ^th^ *Percentile*
Male	9.18	47.43	21.26 – 23.40	11.93	32.4
Female	8.41	55.84	20.50 – 22.95	9.94	36.87
Overall	8.41	55.08	21.26 – 22.79	11.47	36.72

## DISCUSSION

In the present study the mean concentration of zinc was 24.02µmol/L(range 11.4- 36.72). The mean level of zinc which is quoted in reference books is 15µmol/L. This mean level of zinc is lower as compared to our mean level of zinc.^9^ Our study showed a significant difference *p*-value = 0.001 in mean concentration of zinc when compared with mean level of zinc as quoted in reference books.

Reference range of a clinical chemistry parameter is a set of values used in the interpretation of a clinical chemistry report.^12^ Reference interval is defined as a range comprising between 2.5^th^ & 97.5^th^ percentile of the data distribution from a given reference population.^13^

Establishing a normal range of trace elements in the sera of healthy individuals in any geographical area is very important for interpretation of trace element results.^14^ By definition trace elements are normally present in a very low concentration in body.^15^ Zinc is an essential trace element as it is required in our body for performing major functions. Zn plays an important role in immunity, wound healing, growth, reproduction & metabolism of protein & carbohydrate.^16^^,^^17^

Another study carried out in Islamabad determined zinc level & reported to be much higher than the population in Lahore. The mean value of zinc in people living in Islamabad was 126.99µmol/L. This large difference compared to our value might be due to small number of population under study. However the age range in this study was broader as compared to our study. This study showed that geographical area & the diet of the people living in Islamabad/Rawalpindi is main contributor to this difference of mean levels.^18^

In Pakistan, there are several potential sources to contaminate drinking water thus increasing the zinc concentration. Bacteriological contamination of drinking water has been reported to be one of the most serious problems throughout the country in rural as well as urban areas.^19^ Such contamination was attributed to leakage of pipes, pollution from sewerage pipes due to problem within the distribution system, intermittent water supply and shallow water tables due to human activities resulting in increased zinc level.

In addition, excessive monsoon rains, floods, herbicides, fungicides, untreated municipal waste, sewage breakdowns, waste discharges and oil spills, extremely hazardous for drinking water, are constantly being added to the zinc level.

Mushtaq and Khan in 2010 demonstrated that there is heavy metal contamination in soil in response to waste water irrigation in Rawalpindi region. That might be the reason that the people living in Rawalpindi had high levels of zinc in blood.^20^

Parizadeh SM et al in 2011 demonstrated that a low zinc status was a common feature in Persian population. The mean value for zinc was 11.7µmol/L which was lower than our population. This difference might be explained by difference in dietary intake & age of the population under study.^21^

The results of this study are comparable to the study carried out in Austria which determined reference value of zinc in sera of adults. The mean value for zinc in people of Austria with mean age of 25 years was 24.9µmol/L. This value of serum zinc was comparable to our population in Lahore where mean serum zinc concentration was 24.02µmol/L. This high level of zinc was mainly attributed to the geographical region as Austria is rich in lead & zinc ores. Excessive amount of zinc present in environment of Austria might resulted in raised level of zinc in people living in that particular region.^22^

A study conducted in Italy showed a mean concentration of 12.39 µmol/L for zinc which is lower in the population under study. In fact it was expected that there are many factors including age, sex, habits, living standards, working environments & pattern of diseases that results in different levels of zinc in this population.^23^

Zinc is an essential component of all foods & drinking water. The dissolution of zinc from water supplies pipes could be one of the causes of increased concentration of zinc in tap water in Pakistan.^24^

The mean concentration of zinc in our study was lower than the different studies carried out in China, Canada, Italy, Bangladesh, Japan & Spain.^18^ The mean serum zinc concentration of our study compared with various countries is shown in Table-II.

**Table-II:** Mean serum concentration of Zinc in mol/L in various countries

Pakistan	24.2
Austria	24.9
Italy	13.39
Iran	11.7

## CONCLUSION

This study provides data for the establishment of reference range for zinc in healthy population of Lahore. The mean concentration of zinc is 24.02±7.03µmol/L. The results here shows that there is significant difference in serum levels of this element among different countries. This difference may be due to racial and genetic difference, dietary habits and socioeconomic and analytical variables. These values can be useful for interpretation and clinical management of zinc disorders. As there are no established reference ranges for trace elements, these findings can form the basis and reference for any future studies on trace elements in Pakistan.

## Authors Contribution:

Warda Hussain: Conceived the study design, Analysis and interpretation of data and writing of manuscript. Asim Mumtaz: Supervised the study. Farzana Yasmeen: Data collection and analysis of data. Sana Qayyum Khan: Literature Search and interpretation of data. Toqeer Butt: Critical Review.

## Notes

***Source of funding:*** University of Health Sciences, Lahore, Pakistan.
